# Multi-omics approaches to deciphering complex pathological mechanisms of migraine: a systematic review

**DOI:** 10.3389/fphar.2024.1452614

**Published:** 2025-01-09

**Authors:** Jiaojiao Liu, Qiaosheng Ren, Boxuan Du, Xian Liu, Yuqiu An, Peichi Zhang, Lexi Li, Zhenhong Liu, Kegang Cao

**Affiliations:** ^1^ Department of Neurology, Dongzhimen Hospital, Beijing University of Chinese Medicine, Beijing, China; ^2^ Institute for Brain Disorders, Beijing University of Chinese Medicine, Beijing, China

**Keywords:** multi-omics, migraine, inflammation, oxidative stress, mitochondrial dysfunction, systematic review

## Abstract

**Background:**

Migraine represents a chronic neurological disorder characterized by high prevalence, substantial disability rates, and significant economic burden. Its pathogenesis is complex, and there is currently no cure. The rapid progress in multi-omics technologies has provided new tools to uncover the intricate pathological mechanisms underlying migraine. This systematic review aims to synthesize the findings of multi-omics studies on migraine to further elucidate the complex mechanisms of disease onset, thereby laying a scientific foundation for identifying new therapeutic targets.

**Methods:**

We conducted a comprehensive systematic review, specifically focusing on clinical observational studies that investigate various aspects of migraine through the integration of genomics, transcriptomics, proteomics, and metabolomics. Our search encompassed multiple databases including PubMed, EMBASE, the Web of Science Core Collection, the Cochrane Library, China National Knowledge Infrastructure, the Chinese Science and Technology Periodical Database, the Wanfang database, and the China Biology Medicine Database to cover studies from database inception until 20 March 2024., The scope of our review included various aspects of migraine such as ictal and interictal phases; episodic or chronic migraine; menstrual-related migraine; and migraine with or without aura (PROSPERO registration number: CRD42024470268).

**Results:**

A total of 38 studies were ultimately included, highlighting a range of genetic variations, transcriptional abnormalities, protein function alterations, and disruptions in metabolic pathways associated with migraine.These multi-omics findings underscore the pivotal roles played by mitochondrial dysfunction, inflammatory responses, and oxidative stress in the pathophysiology of migraine.

**Conclusion:**

Multi-omics approaches provide novel perspectives and tools for comprehending the intricate pathophysiology of migraine, facilitating the identification of potential biomarkers and therapeutic targets.

**Systematic Review Registration:**

https://www.crd.york.ac.uk/PROSPERO/display_record.php?RecordID=470268, identifier CRD42024470268.

## 1 Introduction

Migraine is a common neurological disorder, clinically characterized by recurrent, often unilateral, moderate to severe pulsating headaches, frequently accompanied by symptoms such as nausea, vomiting, photophobia, and phonophobia (Headache Classification Committee of the International Headache Society IHS, 2018). Based on the characteristics of attacks and remissions, migraine can be classified into the migraine ictal phase (MICP) and the migraine interictal phase MINP). Depending on the presence or absence of aura preceding the headache, migraine is categorized as migraine with aura (MA) and migraine without aura (MO). In terms of attack frequency, migraine is classified as chronic migraine (CM), defined as having fifteen or more migraine days per month and episodic migraine (EM), defined as having fewer than fifteen migraine days per month. Additionally, migraine can be categorized based on their relationship with the menstrual cycle, such as menstrual-related migraine (MM), defined as menstruating women with migraine with or without aura, occurring on day 1 ± 2 of menstruation in at least two out of three cycles, with additional attacks at other times of the cycle (Headache Classification Committee of the International Headache Society IHS, 2018). Recognized by the World Health Organization as the second leading cause of global disability and the third most common disease, migraine affects approximately 15% of the world’s population (Ashina et al., 2021). The 2019 Global Burden of Disease Study underscores migraine’s position as a major cause of disability, with indirect costs from productivity losses amounting to $39.4 billion annually, highlighting its extensive prevalence and significant impact on public health (2020).

However, there are issues with treatment, including insufficient therapeutic responses, contraindications, and adverse effects. For example, new drugs targeting CGRP or its receptor can be used for the acute or preventive treatment of migraine, however, approximately 50% of patients exhibit inadequate responses to these treatments (Wattiez et al., 2020). About 30%–40% of patients experience insufficient efficacy and/or tolerance to triptans, the first-line drugs for acute treatment, Additionally, their use is contraindicated in patients with coronary artery disease and hypertension. Additionally, adverse effects such as fatigue and paresthesia are common (Chinese Medical Association Neurology Branch, 2023). The current treatment landscape remains unsatisfactory, making it crucial to explore the pathogenesis of migraine for improving clinical outcomes.

The pathogenesis of migraine is complex and not yet fully understood, encompassing theories such as the trigeminovascular hypothesis and cortical spreading depression. The development of migraine is closely associated with genetic, endocrine, metabolic, and environmental factors, contributing to its multifaceted etiological framework (Pietrobon and Striessnig, 2003). Previous explorations of migraine mechanisms have largely relied on low-throughput methods such as Polymerase Chain Reaction (PCR) and Enzyme-Linked Immunosorbent Assay (ELISA) to study a limited number of migraine-related biomarkers or to investigate the genetic basis of individual candidate genes and single nucleotide polymorphisms. These approaches fall short in capturing the complex contributions of multiple Pathological factors, offering only a partial explanation of the disease’s associations without comprehensively analyzing or elucidating its underlying mechanisms.

In recent years, the rapid advancement of high-throughput omics technologies has significantly enhanced their application across the biomedical field, establishing them as crucial tools for exploring and deciphering the complex mechanisms of diseases (Hasin et al., 2017). Compared to low-throughput or single-omics approaches, multi-omics research employing high-throughput technologies enables the simultaneous analysis of genes, transcripts, proteins, and metabolites. This integrative approach facilitates a comprehensive and systematic identification of key targets associated with disease onset and progression.

Multi-omics research integrates disciplines such as genomics, transcriptomics, proteomics, and metabolomics (Babu and Snyder, 2023). Techniques such as microarrays, whole genome sequencing, and whole exome sequencing delve into the architecture, functionality, and interactions within the human genome in genomics studies (Lazaridis and Petersen, 2005; Green et al., 2011). Transcriptomics leverages high-throughput methods like gene chip technology and RNA sequencing to thoroughly examine gene transcription and regulatory processes within cells (Wang et al., 2009; Manzoni et al., 2018). In proteomics, mass spectrometry (MS) and two-dimensional gel electrophoresis (2-DE) analyze an organism’ scomplete protein complement, exploring expression, functions, and regulations (Zhu et al., 2003). Metabolomics, through platforms such as gas chromatography-mass spectrometry (GC-MS), liquid chromatography-mass spectrometry (LC-MS), and nuclear magnetic resonance (NMR), quantifies a vast array of metabolites at precise moments, accommodating both targeted and untargeted studies (Rochfort, 2005). Integrating results from these diverse omics fields not only enhances the robustness and credibility of the research findings but also significantly deepens our understanding of the complex pathological mechanisms underlying migraine. This holistic approach is crucial for identifying biomarkers associated with migraine and advancing the effectiveness of migraine treatments.

Multi-omics research incorporates comprehensive studies on both human and animal models, providing essential insights into the mechanisms of migraine. However, clinical studies are generally considered to provide stronger evidential support than animal experiments due to their direct applicability to human biology. Moreover, the distinctive characteristics of various migraine subtypes are often not replicable in animal models, limiting their utility in mimicking the full spectrum of the disorder. Consequently, this paper exclusively includes clinical observational studies of common migraine subtypes, employing a multi-omics approach to provide a systematic overview. To our knowledge, this systematic review is the first to categorize common subtypes of migraine and integrate clinical observational studies concerning multi-omics, offering a comprehensive and systematic assessment of the multi-omics characteristics of migraine.

## 2 Methods

The current systematic review was conducted following the Preferred Reporting Items for Systematic Reviews and Meta-Analyses (PRISMA) guidelines. The protocol was registered on the International Prospective Register of Systematic Reviews (PROSPERO) with the registration number CRD42024470268 (https://www.crd.york.ac.uk/PROSPERO/display_record.php?RecordID=470268).

### 2.1 Search strategy and selection criteria

The systematic search for migraine-related studies encompassed multiple databases, including PubMed, EMBASE, the Web of Science Core Collection, the Cochrane Library, China National Knowledge Infrastructure, the Chinese Science and Technology Periodical Database, the Wanfang database, and the China Biology Medicine Database. Searches were conducted in both English and Chinese, covering records from the inception of each database up to 20 March 2024. The search strategy utilized both MeSH terms and free-text terms, with details provided in Supplementary Table S1.

The inclusion criteria are outlined as follows: 1) participants diagnosed with migraine, irrespective of age, gender, or severity, encompassing various subtypes such as MA and MO, CM and EM, MICP and MINP, and MM; 2) studies employing omics techniques, including genomics, proteomics, metabolomics, and transcriptomics, to investigate migraine mechanisms; 3) Clinical observational study in which the control group consisted of healthy individuals with no history of migraines or headaches, matched by age and gender to the migraine group. Exclusion criteria are defined as follows: 1) studies presented in formats such as abstracts, editorials, letters, commentaries, case reports, experimental research, and review articles; 2) studies on patients with primary headache types other than migraine or secondary headaches caused by conditions such as hypertension or brain tumors; 3) studies where the control groups consist of non-healthy individuals.

Two researchers (QR and BD) independently screened the literature. Initially, they swiftly excluded documents that did not meet the requirements by reviewing titles, keywords, and abstracts. Subsequently, the researchers conducted a detailed examination of the remaining articles to confirm their compliance with the inclusion criteria and performed a cross-verification of the results to further exclude any that did not meet the standards. In case of any disagreements, consensus will be reached, or any disputes will be resolved through consultation with a third researcher (JL).

### 2.2 Data extraction

Data were extracted independently by two researchers (XL and QA). Relevant information from the included documents, such as literature name, study location, study design type, migraine subtype, sample size, type of omics used, omics techniques, specimens, and main findings, was recorded using Excel. The extracted data underwent cross-verified for accuracy. In case of disagreement, a third researcher (JL) was consulted, and final decision were made collaboratively.

### 2.3 Quality assessment

Two independent researchers (LL and PZ) conducted quality assessments of case-control studies using the Newcastle-Ottawa Scale, which consists of three dimensions and eight items. Scores range from 0 to 9 points, with higher total scores indicating better study quality. Concurrently, quality assessments for cross-sectional studies were conducted using the standards recommended by the Agency for Healthcare Research and Quality, which involve 11 criteria. Total scores range from 0 to 11 points, where studies scoring ≤5 are considered low quality, scores of 6–7 are deemed medium quality, and scores ≥8 indicate high quality. In case of disagreements, consensus was reached through discussion with a third researcher (JL), ensuring agreement on the quality evaluation.

## 3 Results

**FIGURE 1:**
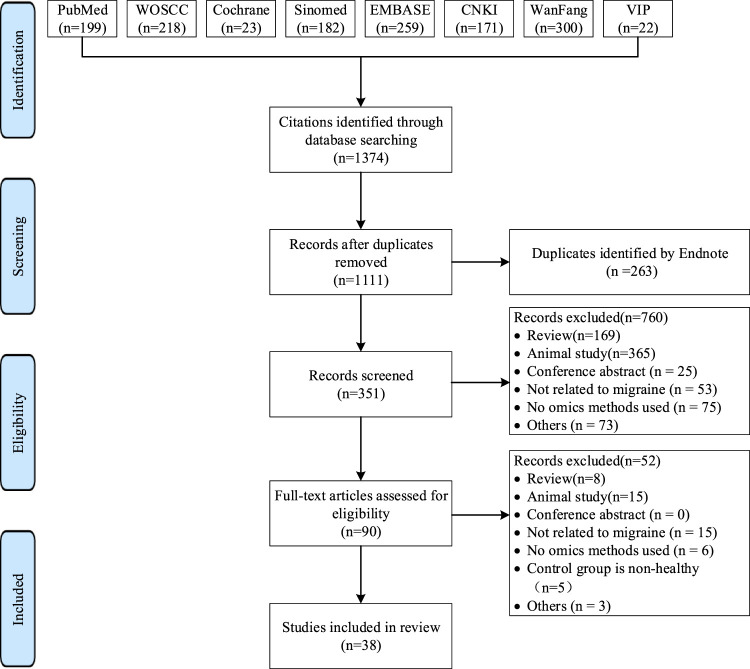
Study Selection Flowchart, modified from the PRISMA 2020 flow diagram template.WOSCC, Web of Science Core Collection; SinoMed, China Biology Medicine Database; CNKI, China National Knowledge Infrastructure; VIP, Chinese Science and Technology Periodical Database.

A systematic search of eight databases yielded 1,347 articles. After reviewing titles, abstracts, and full texts, 38 articles were ultimately included. The literature selection process is illustrated in Figure 1 (Hershey et al., 2012 ^;^ Gerring et al., 2018; Hershey et al., 2004; Al Asoom et al., 2022; Chasman et al., 2011; Freilinger et al., 2012; Chen et al., 2017; Khan et al., 2022; Nagata et al., 2009; Kogelman et al., 2019; Carreño et al., 2011; Aczél et al., 2022; Lin et al., 2020; Gallardo et al., 2023; Andersen et al., 2016; Pan Jianqing and Wu, 2019; Togha et al., 2022; Bellei et al., 2021; Bellei et al., 2020; Guyuron et al., 2014; Xu et al., 2022; Ren et al., 2018a; Curto et al., 2015; Rustichelli et al., 2021; Harder et al., 2021; Onderwater et al., 2019; Aczél et al., 2021; de Tommaso et al., 2012; Welch et al., 1989; Onderwater et al., 2023; Rustichelli et al., 2020; D'Andrea et al., 2022; Castor et al., 2021; Peterlinet al., 2015; Ren et al., 2018b; Gouveia-Figueira et al., 2017; Tuka et al., 2021; Curto et al., 2015).

In terms of migraine diagnosis, 30 studies relied on diagnoses made by clinicians and 8 studies did not specify the diagnostic approach in detail. For the setup of the healthy control group, 12 studies explicitly defined healthy controls as individuals without any history of headaches, 10 studies specifically defined controls as individuals without a history of migraines, and 16 studies did not clearly define the criteria for healthy controls. Regarding gender distribution, 10 studies exclusively included female subjects. Among these, 4 studies focused on menstrual-related migraine, with 1 study in genomics, 2 in proteomics, and 1 in metabolomics. The remaining 28 studies included both male and female subjects, with a predominance of female participants. Concerning the origin of study participants, 26 studies were conducted in Western countries, 7 studies were from East Asian countries, and the remaining 5 studies were from the Middle East and Oceania regions. In terms of study design types and quality assessment, eight studies employed a cross-sectional design. Among these, one study received a quality assessment score of 6 points, indicating moderate quality, while seven studies scored between 3 and 5 points, indicating low quality. Additionally, thirty-one studies utilized a case-control design. Of these, eight studies had quality assessment scores ranging from 6 to 7 points and twenty-three studies scored between 1 and 5 points, indicating low quality. Overall, the majority of the included studies were of moderate to low quality.The quality assessment results of the included literature are provided in Supplementary Tables S2, S3. In terms of omics research types, thirteen studies reported genomic characteristics of migraine; eight studies described transcriptomic features; six studies detailed proteomic traits; and seventeen studies revealed metabolic characteristics associated with migraine. Distribution of multi-omics studies among migraine subtypes included six in either MICP and MICP, sixteen in MA and MO, eight in CM and EM, four in MM, and nine multi-omics studies that met the migraine diagnosis criteria but did not specify subtypes. This review categorizes the findings according to omics categories and migraine subtypes. Studies without specifying subtypes are presented separately in a section for non-subtyped migraine (NSM).

### 3.1 Genomic studies on migraine

This section encompasses thirteen studies, comprising five studies on MA and MO, one study on CM and EM, two on MM, and four studies where the migraine subtype was not specified. These studies employed genomic techniques such as gene sequencing, microarrays, or genotyping, utilizing whole blood (WB) or peripheral blood lymphocytes (PBL) as samples for genomic analysis. Details on the included studies are provided in Table 1.

**TABLE 1:** Summary of principal outcomes in genomic studies.

Source	Study design	Observation group		Control group		Specimen	Technology	Main findinngs
		Disease	Sample size	Type	Sample size			
America Hershey et al. (2012)	case control	MM	18	H/No history of headaches	18	WB	microarray	MM is characterized by 77 specific genes, primarily involved in biological pathways related to mitochondrial function, oxidative phosphorylation, and metal ion binding
Australia; Gerring et al., 2018	case control	NSM	67	H/No history of migraines	67	PBL	DNA methylation arrays	Migraine involves 62 differentially methylated regions linked to regulatory elements and genes related to solute transport and hemostasis
America Hershey et al. (2004)	case control	EM/CM	7/15	H	13	WB	microarray	CM patients show 353 upregulated genes linked to mitochondrial dysfunction; EM involves 40 upregulated genes related to inflammation and gene regulation changes
Saudi Arabia Al Asoom et al. (2022)	case control	NSM	20	H	20	WB	Next-generation Sequence	30 mitochondrial SNPs significantly associated with migraine suggest mechanisms linked to mitochondrial dysfunction
America, Germany, Netherlands Chasman et al. (2011)	case control	NSM	5122	H/No history of migraines	18,108	WB	microarray	The variants PRDM16、TRPM8 and LRP1 are migraine susceptibility loci linked to neurotransmitter pathways and pain-related processes
Germany, Netherlands Freilinger et al. (2012)	case control	MO	2326	H/No history of migraines	4580	WB	microarray	MEF2D, TGFBR2, PHACTR1, and ASTN2 are migraine susceptibility loci, closely related to neuronal excitability and vascular function mechanisms
China Chen et al. (2017)	case control	MO	1005	H/No history of migraines	1053	NA	microarray	4 genetic loci associated with MO: DLG2, GFRA1, TRPM8, and LRP1, involving glutamatergic neurotransmission and trigeminovascular system function
Spain Khan et al. (2022)	case control	NSM	20	H/No history of migraines	20	WB	Whole-Exome Sequence	17 functional variants in 12 genes significantly increased migraine risk, involving neurological, inflammatory, and cellular stress responses
Arab Nagata et al. (2009)	case control	MA	7	H	7	PBL	microarray	15 genes were significantly differentially expressed in patients with MA, with one-fifth of these genes related to cytoskeletal proteins
Japan Kogelman et al. (2019)	case control	MA/MO	9/17	H/No history of headaches	20	WB	RNA-Seq	No significant gene expression differences were found between migraine patients and healthy controls
Denmark; Carreño et al., 2011	case control	MA/MO	1040	H/No history of headaches	1037	PBL	SNP genotyping	Nineteen SNPs in ten TRP genes were significantly linked to MO and MA, especially rs7217270 in TRPV3 and rs222741 in TRPV1, which positively correlated with migraine incidence

#### 3.1.1 Migraine with or without aura

Patients with MA have 15 specific genes primarily involved in cytoskeletal structure, calcium ion binding, neurotransmitter synthesis and metabolism, and oxidative phosphorylation pathways (Nagata et al., 2009). The susceptibility genes for MO include myocyte enhancer factor 2D, transient receptor potential melastatin 8, and low-density lipoprotein receptor-related protein 1 (Freilinger et al., 2012; Chen et al., 2017), which are associated with mechanisms of neuronal excitability and synaptic regulation, cell proliferation and differentiation, synaptic activity, and endothelial function.

There are also shared susceptibility genes between MA and MO. For example, 19 SNPs in 10 genes within the transient receptor potential superfamily of non-selective cation channels are significantly associated with both MO and MA. These differential genes are related to cytoskeletal proteins and neural plasticity, potentially playing a role in neuronal excitability and synaptic regulation (Carreño et al., 2011). However, some studies indicate that there are no significantly differentially expressed genes when comparing patients with MA and MO to healthy controls (Kogelman et al., 2019).

#### 3.1.2 Chronic migraine and episodic migraine

Compared to healthy controls, patients with CM and EM exhibit upregulated expression of platelet-related genes, including Clusterin, SPARC, and Neurogranin, indicating signs of platelet activation and dysfunction. Among these, EM patients show higher expression of the c-fos and cox-2 genes, while CM patients exhibit elevated expression of specific mitochondrial genes. This suggests that inflammation may play a critical role in EM, whereas mitochondrial dysfunction might be pivotal in the pathophysiology of CM (Hershey et al., 2004).

#### 3.1.3 Menstrual-related migraine

MM patients exhibit 77 unique genes and these genes are primarily associated with immune regulation, inflammation response, mitochondrial function, and DNA homeostasis (Hershey et al., 2012).

#### 3.1.4 Migraine without subtype classification

Migraine patients exhibit 62 distinct differentially methylated regions, which are closely associated with genes involved in solute transport and coagulation processes. Additionally, three significant susceptibility loci, rs2651899 (PRDM16), rs10166942 (TRPM8), and rs11172113 (LRP1), are significantly associated with migraine and are related to pain and neurotransmitter pathways, supporting the pathophysiological perspective of migraine ^(^ Gerring et al., 2018; Chasman et al., 2011).Furthermore, whole-exome sequencing has identified 17 functional variants in 12 genes, including RETNLB and ADH4, that are closely linked to the occurrence of migraine (Khan et al., 2022). Mitochondrial DNA variations are also present in migraine patients, predominantly involving 30 SNPs in nine genes such as COX2, COX1, and COX3, which encode key proteins of the mitochondrial respiratory chain complex, highlighting the potential role of mitochondrial dysfunction in migraine pathogenesis (Al Asoom et al., 2022).

### 3.2 Transcriptomic studies on migraine

In this section, six studies are included, comprising one study on MO or MA, three investigations of MICP and MICP, and two studies on NSM. Transcriptomic analyses were conducted using RNA sequencing or microarray technologies, with samples derived from whole blood, serum, plasma, or peripheral blood mononuclear cells. Details of the included studies are provided in Table 2.

**TABLE 2:** Summary of principal outcomes in transcriptomics studies.

Source	Study design	Observation group		Control group		Specimen	Technology	Main findinngs
		Disease	Sample size	Type	Sample size			
Hungary Aczél et al. (2021)	case control	MICP/MINP	3/21	H	13	PBL	RNA sequence	During MICP, 131 genes are differentially expressed in inflammatory pathways and cytokine activity. In MINP, 163 genes show changes in inflammatory response and mitochondrial oxidative phosphorylation
Hungary Aczél et al. (2022)	case control	MICP/MINP	16	H	12	PBL	small RNA sequence	In MINP, 31 miRNAs were differentially expressed, involving immune responses, inflammation, and oxidative stress. In MICP, 31 miRNAs differed, mainly related to immune and inflammatory pathways
China Lin et al. (2020)	case control	NSM	4	H	3	plasma	circRNA microarray	2,039 circRNAs exhibited differential expression, involving cellular signaling and inflammatory responses
Spain Gallardo et al. (2023)	case control	NSM	20	H/No history of headaches	12	PBL	miRNA microarray	191 miRNAs showed differential expression, involving ion channels, neurotransmitters, and hormones
Denmark; Andersen et al., 2016	case control	MICP/MINP	8	H	8	serum	miRNA arrays	During MICP, 32 miRNAs showed abnormal expression linked to inflammatory genes. Notably, miR-382-5p was elevated in both MICP and MINP, potentially serving as a chronic biomarker for migraine
China Pan Jianqing and Wu (2019)	case control	MO	5	H	5	WB	BGISEW-500 sequence	MO patients show 821 differentially expressed miRNAs, impacting axon development, neuron processes, and cell adhesion

#### 3.2.1 Migraine without aura

Compared to healthy controls, patients with MO exhibit 821 differentially expressed miRNAs. The target genes of these miRNAs are primarily involved in axon development, neuron-related pathology processes, and cell signaling pathways (Pan Jianqing and Wu, 2019).

#### 3.2.2 Migraine attack and interictal phases

Transcriptomic studies during migraine attacks indicate that, compared to healthy controls, migraine patients exhibit 144 differentially expressed genes. Notably, genes such as IL1β, COX2, TNF, and IL8 are significantly upregulated, which are primarily involved in pathology pathways related to inflammatory responses, cytokine activity, and mitochondrial dysfunction ^(^ Aczél et al., 2021 ^)^. Additionally, among the 372 miRNAs detected in migraine patients, about 8% are abnormally expressed. miR-34a-5p and miR-382-5p show the most significant increase during migraine attacks, which may be associated with neuroinflammation and the activation of pain transmission pathways (Andersen et al., 2016) Other studies targeting miRNAs during the migraine MINP indicate 31 differentially expressed miRNAs are identified, which are mainly related to immune response, neuroinflammation, and oxidative stress (Aczél et al., 2022). Transcriptomic studies during MINP reveal 163 differentially expressed genes compared to healthy controls, with IL1B, PTGS2, TNF, and IL8 being the most significantly upregulated (Aczél et al., 2021), suggesting that inflammatory activity may persist even during the interictal period. Furthermore, 31 differentially expressed miRNAs detected during the remission phase are closely related to immune regulation, neuroinflammation, stress response, and cellular protective processes (Aczél et al., 2022).

#### 3.2.3 Migraine without subtype classification

Compared to healthy controls, migraine patients exhibit 2039 specific circRNAs, with significant enrichment in the PI3K-Akt signaling pathway. This suggests that these circRNAs may promote inflammatory responses by regulating this pathway, thereby driving the occurrence of migraine (Lin et al., 2020). Moreover, transcriptomic profiling of miRNAs revealed 191 differentially expressed miRNAs, which are primarily linked to ion channels, particularly calcium channels, and key signaling pathways such as TRP, HIF-1, TGF-β, GnRH, and Notch. They also relate to neurotransmitters like serotonin, dopamine, and adrenaline, as well as hormone regulation including aldosterone and growth hormone (Gallardo et al., 2023).

### 3.3 Proteomic studies on migraine

In this section, a total of five studies are included. These include one study on MA and MO, one study on MICP and MINP, two studies on MM, and one study on NSM. Proteomic analysis was performed on migraine patients’ serum, plasma, urine, or tissue from the zygomaticotemporal branch of the trigeminal nerve after migraine surgery, using techniques such as MS, 2-DE combined with MS, or liquid chromatography-tandem mass spectrometry (LC-MS). Details of the included studies are provided in Table 3.

**TABLE 3:** Summary of principal outcomes in proteomics studies.

Source	Study design	Observation group		Control group		Specimen	Technology	Main findinngs
		Disease	Sample size	Type	Sample size			
Iran Togha et al. (2022)	case control	MICP/MINP	23/35	H	29	plasma	2-DE/LC-MS/MS	The differential proteins are associated with inflammation, oxidative stress, immune response, and neuroprotection
Italy Bellei et al. (2021)	cross-sectional	MM	15	H/No history of headaches	15	Urine	2DE/LC-MS/MS	21 urinary proteins showed abnormal expression, involving immune and inflammatory responses and neuroprotection
Italy Bellei et al. (2020)	case control	MM	15	H/No history of headaches	15	serum	LC-ESI-QToF-MS/MS/2-DE	In MM patients, 45 proteins were differentially expressed, involving inflammation, cell signaling, and immune regulation
America Guyuron et al. (2014)	case control	NSM	15	H/No history of migraines	15	Trigeminal Nerve	LC-MS	173 proteins were differential expression, involving in oxidative stress, membrane function, and neuroprotective mechanisms
China; Xu et al., 2022	case control	MA/MO	20	H/No history of headaches	20	serum	MALDI-TOF-MS	Significant differences in the glycosylation levels of IgG1 G1, IgG2 G0, and IgG2 G0N relate to immune response and inflammation and no significant glycosylation changes were observed in MO.

#### 3.3.1 Migraine with or without aura

Jingwei Xu (Xu et al., 2022) detected 49 types of IgG N-glycopeptides in patients with MA and MO. Among these, the level of the IgG1 G0-NF N-glycopeptide was significantly elevated, suggesting it could be an important biomarker for diagnosing migraine. This indicates a potential relationship between migraine and immunomodulation.

#### 3.3.2 Migraine attack and interictal phases

During both MICP and MINP, compared to healthy controls, the expression of proteins such as transthyretin, haptoglobin, clusterin, α1-microglobulin, and retinol-binding protein 4 is significantly increased in plasma. These differentially expressed proteins are closely associated with inflammatory responses, oxidative stress, and neuroprotection processes, highlighting their important role in the pathophysiology of migraine (Togha et al., 2022).

#### 3.3.3 Menstrual migraine

Urinary proteomic profiling in patients with MM identifies 21 significantly modulated proteins (Bellei et al., 2021), including s100A8, kininogen-1, albumin and immunoglobulin heavy constant gamma 2, primarily involved in inflammation and immune response mechanisms. In contrast, serum proteomic analysis showed disparities in 12 proteins compared to controls, highlighting increased levels of complement c4-A and the inflammatory marker ITIH4, with a decrease in the anti-inflammatory apolipoprotein A-I, elucidating pathways in anti-inflammation, vascular repair, and neuroprotection (Bellei et al., 2020).

#### 3.3.4 Migraine without subtype classification

Comparative proteomic analysis reveals 173 proteins significantly differentially expressed in migraine patients relative to healthy controls. Key proteins such as MYH9, PRDX2, AHNAK, STOM, and PARK7 are predominantly associated with oxidative stress response, cellular membrane functionality, and neuroprotection. The ephrin-B signaling pathway exhibits the most substantial differential expression (Guyuron et al., 2014).

### 3.4 Metabolomics studies on migraine

This section encompasses 17 studies, including seven studies on MA and MO, four on CM and EM, three during MICP and MINP, and three on NSM. These studies employed techniques such as GC-MS, LC-MS, or NMR, using cerebrospinal fluid, serum, or plasma samples for metabolomic analysis. Details of the included studies are provided in Table 4.

**TABLE 4:** Summary of principal outcomes in metabolomics studies.

Source	Study design	Observation group		Control group		Specimen	Technology	Main findinngs
		Disease	Sample size	Type	Sample size			
Netherlands Zielman et al. (2016)	case control	MA/MO	27/43	H/No history of headaches	43	CSF	1H-NMR	Migraine patients showed no distinct metabolic profile compared to healthy controls
China Ren et al. (2018a)	case control	MO	20	H/No history of migraines	20	serum	LC-MS	In MO, 29 significant lipid metabolites, including Cer_NSs, lysoPCs, and lysoPEs, involved in oxidative stress and intracellular signaling
Italy Curto et al. (2015)	case control	CM	119	H	84	serum	LC-MS	Nnine metabolites showed significant differences, including reductions in KYN, KYNA, and 3-HK, suggesting increased neural excitability
Italy Rustichelli et al. (2021)	case control	MO	30	H/No history of headaches	30	serum	LC-MS/MS	Levels of DHEAS, DHEA, and DHP are significantly reduced, indicating insufficient neuroprotection, anti-inflammatory activity, and pain modulation
Netherlands Harder et al. (2021)	case control	NSM	313	H/No history of migraines	1512	serum	1H-NMR	28 metabolites associated with migraine involve lipid, glucose, and amino acid pathways, involving energy metabolism, inflammation, cell membrane function, and neurotransmission
Netherlands Onderwater et al. (2019)	case control	NSM	2800	H/No history of migraines	7353	plasma	1H-NMR	Apolipoprotein 1 levels, free cholesterol/total lipids ratio in small HDL, and omega-3 fatty acids (in males) decreased, confirming HDL changes associated with migraine
Hungary Aczél et al. (2021)	case control	MICP/MINP	3/21	H/No history of headaches	13	plasma	LC-MS/MS	In MICP, succinic acid and methionine sulfoxide increase, spermine and spermidine decrease, indicating mitochondrial dysfunction and oxidative stress. In MINP, elevated lactate and succinic acid suggest mitochondrial dysfunction
Italy; de Tommaso et al., 2012	case control	MA/MO	147	H	34	serum	LC-MS	In MA, significantly lower N-acetyl-aspartate levels suggest impaired neuronal mitochondrial function. No specific metabolite differences were noted for migraine without aura
Italy Welch et al. (1989)	cross-sectional	MICP/MINP	12/8	H	27	cortex	31 NMR	In MICP, the ratio of PCr to Pi decreases, indicating reduced mitochondrial energy metabolism; no significant changes in the PCr/Pi ratio were found In MINP.
Netherlands Onderwater et al. (2023)	case control	MINP	96/98	H	96	CSF and plasma	UPLC-MS	Reduced L-arginine levels in the CSF of migraine patients during MINP indicate a dysfunction in nitric oxide
Italy Rustichelli et al. (2020)	cross-sectional	MM	30/30	H/No history of headaches	60	serum	HPLC-ESI-MS/MS	Significantly lower levels of allopregnanolone may lead to increased neuronal excitability
Italy D'Andrea et al. (2022)	case control	CM	37	H	21	plasma	UPLC–ESI–MS/MS	CM sufferers show lower arginine and higher ornithine, ADMA, and NMMA levels, indicating inhibited nitric oxide synthesis
America Castor et al. (2021)	case control	CM	15	H/No history of headaches	10	serum and csf	GC-MS/LC-MS/MS	Elevated saturated, monounsaturated, and polyunsaturated fatty acids indicate lipid metabolism abnormalities linked to energy imbalances, inflammation, and insulin resistance
America Peterlinet al. (2015)	cross-sectional	EM	52	H/No history of headaches	36	serum	HPLC-MS/MS	Lower total ceramides and dihydroceramides but higher C18:0 and C18:1 sphingolipids, involving energy, apoptosis, and inflammation
China Ren et al. (2018b)	case control	NSM	20	H/No history of migraines	20	serum	LC-MS	10 metabolites, including serotonin and amino acids, are significantly reduced, impacting tryptophan, arginine, proline metabolism, and aminoacyl-tRNA biosynthesis
Sweden Gouveia-Figueira et al. (2017)	case control	MA/MO	26/122	H	26	plasma	LC-MS	No significant differences in AEA, NAEs, or linoleic acid-derived oxylipins between migraine patients and controls
Hungary Tuka et al. (2021)	case control	MICP/MINP	47/12	H	34	CSF	1H-NMR	Migraine patients showed no distinct metabolic profile compared to healthy controls
Italy Curto et al. (2015)	case control	CM/EM	50/50	H/No history of migraines	50	serum	LC-MS	In MO, 29 significant lipid metabolites, including Cer_NSs, lysoPCs, and lysoPEs, involved in oxidative stress and intracellular signaling

#### 3.4.1 Migraine with or without aura

In patients with MO, 29 lipid metabolites in the serum are significantly altered. Among these, Cer_NS levels are significantly elevated, and lysoPE levels are significantly reduced, showing the most notable changes. These abnormal lipid metabolites primarily regulate pathology processes such as inflammation, oxidative stress, and cell signal transduction (Ren et al., 2018a). Additionally, serum levels of neurosteroids such as dehydroepiandrosterone sulfate, dehydroepiandrosterone, and 5α-dihydroprogesterone are significantly lower in patients with MA compared to healthy controls. These abnormal neurosteroid levels support the notion that migraine patients may have insufficient neuroprotective and anti-inflammatory activities, as well as impaired pain regulation (Rustichelli et al., 2021).

In patients with MA, serum N-acetylaspartate levels are significantly lower than those in healthy controls, suggesting that low NAA levels may be a marker of migraine-related neurological dysfunction, potentially due to mitochondrial dysfunction (de Tommaso et al., 2012).

Other studies have indicated that there are no significant differences in metabolites between MA and MO and healthy controls. For example, Gouveia-Figueira’s (Gouveia-Figueira et al., 2017) research found no significant differences in 29 plasma lipid metabolites, including endocannabinoids and N-acylethanolamines, between migraine patients and healthy controls. Similarly, Ronald Zielman (Zielman et al., 2016) found no significant differences in cerebrospinal fluid metabolites between migraine patients and healthy controls.

#### 3.4.2 Migraine attack and interictal phases

During MICP, patients exhibit significantly increased plasma levels of methionine sulfoxide and succinate, while levels of aconitate, spermine, and spermidine are significantly decreased. These abnormalities are mainly associated with mitochondrial dysfunction and oxidative stress (Aczél et al., 2021). Additionally, plasma levels of anthranilic acid, 5-hydroxyindoleacetic acid, and melatonin are significantly elevated, which may represent compensatory responses to pain attacks (Tuka et al., 2021). Using phosphorus-31 nuclear magnetic resonance (31P NMR) technology, a decrease in the molar percentage of phosphocreatine (PCr) and an increase in the molar percentage of inorganic phosphate (Pi) have been revealed in the frontal cortex, frontotemporal cortex, occipitoparietal cortex, and occipital cortex, indicating abnormal brain energy metabolism during MICP (Welch et al., 1989).

During MINP, patients exhibit abnormal changes in metabolites in the plasma, cerebrospinal fluid, and specific cortical regions. Plasma levels of lactate and succinate are significantly elevated, primarily indicating mitochondrial dysfunction and energy metabolism imbalance (Aczél et al., 2021). Meanwhile, levels of transient receptor potential, L-kynurenine, 5-hydroxyindoleacetic acid, and melatonin are significantly reduced, suggesting inhibited tryptophan metabolism, with excessive glutamate causing neurotoxicity and hyperexcitability, thereby increasing migraine susceptibility (Tuka et al., 2021). L-arginine levels are decreased in the cerebrospinal fluid but unchanged in the plasma, indicating that L-arginine metabolism may be affected only centrally rather than systemically (Onderwater et al., 2023). In the frontal and frontotemporal regions, the PCr/Pi ratio and PCr/total phosphorus ratio are reduced, while the Pi/total phosphorus ratio is increased, indicating abnormal brain energy metabolism even during MINP (Welch et al., 1989).

#### 3.4.3 Chronic migraine and episodic migraine

In patients with CM, significant changes occur in tryptophan metabolites such as KYN, KYNA, and 3-HK. These changes may be related to the regulation of NMDA receptor activity and pain transmission in the central nervous system (Curto et al., 2015). Similarly, plasma metabolomics indicate decreased levels of arginine and increased levels of ornithine, ADMA, and NMMA, suggesting inhibited arginine metabolism and potential defects in the nitric oxide synthesis and release pathway (D'Andrea et al., 2022).Concurrent analysis of lipid metabolism in cerebrospinal fluid and plasma of CM reveals higher levels of 5 saturated fatty acids, 8 monounsaturated fatty acids, 5 omega-3 polyunsaturated fatty acids, and 5 omega-6 polyunsaturated fatty acids in plasma. The elevation of these unesterified fatty acids may be associated with increased lipid metabolic activity. In cerebrospinal fluid, the esterified D5D index and platelet-activating factor are lower, while the esterified and unesterified levels of the monounsaturated fatty acid C16:1n-7 are higher. Overall, the abnormal lipid metabolism in plasma and cerebrospinal fluid may be related to changes in energy homeostasis, pain regulation, and inflammatory processes (Castor et al., 2021).

In patients with episodic migraine, serum sphingolipid metabolites exhibit changes, primarily characterized by decreased levels of total ceramides and dihydroceramides. These altered sphingolipid metabolites are closely related to energy metabolism, apoptosis, and inflammatory responses (Peterlinet al., 2015).

Both chronic migraine and episodic migraine patients show significantly elevated serum levels of spermine and spermidine, while agmatine levels are significantly reduced in episodic migraine patients. Spermidine and spermine can activate NMDA receptors, whereas agmatine can inhibit NMDA receptor activity. This may explain the hyperactivity of NMDA receptors in the pathogenesis of migraine, providing a potential rationale for the use of NMDA receptor polyamine site antagonists in migraine treatment (Lionetto et al., 2021).

#### 3.4.4 Menstrual migraine

Targeted metabolomics studies on menstrual migraine reveal that serum pregnenolone levels in migraine patients during the follicular phase are significantly lower compared to those in healthy controls. These levels also negatively correlate with the duration of migraine history and the frequency of attacks. Notably, levels of testosterone and progesterone did not show significant differences. The reduction in pregnenolone may lead to decreased GABAergic inhibition and increased cerebral excitability, potentially triggering MM episodes (Rustichelli et al., 2020).

#### 3.4.5 Migraine without subtype classification

In migraine patients, 28 serum metabolites are associated with the migraine state, primarily involving lipid, glucose, and amino acid metabolic pathways. These reflect abnormalities in energy metabolism, inflammatory responses, cell membrane function, and neural conduction pathways (Harder et al., 2021). Additionally, serum levels of serotonin and nine amino acids are significantly reduced. These metabolites are mainly related to tryptophan metabolism, arginine and proline metabolism, and aminoacyl-tRNA biosynthesis pathways (Ren et al., 2018b). Plasma metabolomics in migraine patients indicate decreased levels of apolipoprotein A1, reduced ratios of free cholesteryl esters to total lipids in small HDL subtypes, and significantly decreased omega-3 fatty acids in men only. These findings confirm the association between specific functional alterations in HDL and the migraine state (Onderwater et al., 2019).

### 3.5 Integrated multi-omics analysis

Each type of omics data typically provides a list of disease-related differences independently. However, the analysis of a single omics data type often only reveals correlations, which mostly reflect reactive rather than causative processes. Therefore, integrating multiple types of omics data is crucial for revealing potential causative changes and therapeutic targets in diseases. This section conducts a joint qualitative analysis of multi-omics studies on the same migraine subtype, aiming to delve deeper into the causative changes characteristic of migraine.

Integrated multi-omics analysis is an approach that combines data from different omics layers to achieve a more comprehensive and systematic understanding of biology (Welch et al., 1989; Maghsoudi et al., 2022). Generally, multi-omics integration analysis involves simultaneously acquiring and analyzing various omics data from the same set of samples or the same patient. However, there are currently few studies that perform integrated multi-omics analyses on the same cohort of migraine patients. Among the literature included in this paper, only one study integrates transcriptomics and metabolomics. The core of multi-omics analysis lies in integrating different types of omics data to comprehensively analyze the complexity of Pathological systems and the interrelationships between different layers. Therefore, this paper describes and analyzes the multi-omics results of different migraine patients to comprehensively assess the complexity of the mechanisms underlying migraine pathogenesis.

#### 3.5.1 Migraine with and without aura

Multi-omics studies on MA and MO have elucidated the primary pathomechanisms involved. MA is closely associated with pathology pathways including neurotransmitter synthesis, inflammatory responses, oxidative phosphorylation, immune responses, and mitochondrial dysfunction. Conversely, MO is significantly related to inflammatory responses, neuronal excitability and synaptic regulation, cell proliferation and differentiation, endothelial function, axonal development, neuron-associated pathology processes, as well as cellular signaling, immune modulation, oxidative stress, and signal transduction pathology processes.

#### 3.5.2 Chronic and episodic migraine

Multi-omics studies on CM and EM indicate that mitochondrial dysfunction, alterations in energy homeostasis, nociception regulation, and inflammatory responses are closely associated with CM. In contrast, inflammatory responses, energy metabolism, and apoptosis are significantly related to the occurrence of episodic migraine.

#### 3.5.3 Migraine attack and interictal phases

Multi-omics studies on the MICP and MINP provide evidence supporting the roles of inflammation, mitochondrial dysfunction, oxidative stress, and abnormalities in neuroprotective functions in the pathogenesis of migraine.

#### 3.5.4 Menstrual migraine

Multi-omics studies on MM indicate that immune modulation, inflammatory responses, mitochondrial function and DNA stability, as well as increased brain excitability, play crucial roles in the pathogenesis of migraine.

#### 3.5.5 Migraine without subtype classification

Integrated analysis of multi-omics results for NSM reveals that the occurrence of migraine is closely associated with inflammation, oxidative stress, and mitochondrial dysfunction.

#### 3.5.6 Frequency of multi-omics mechanisms in various migraine subtypes

**FIGURE 2:**
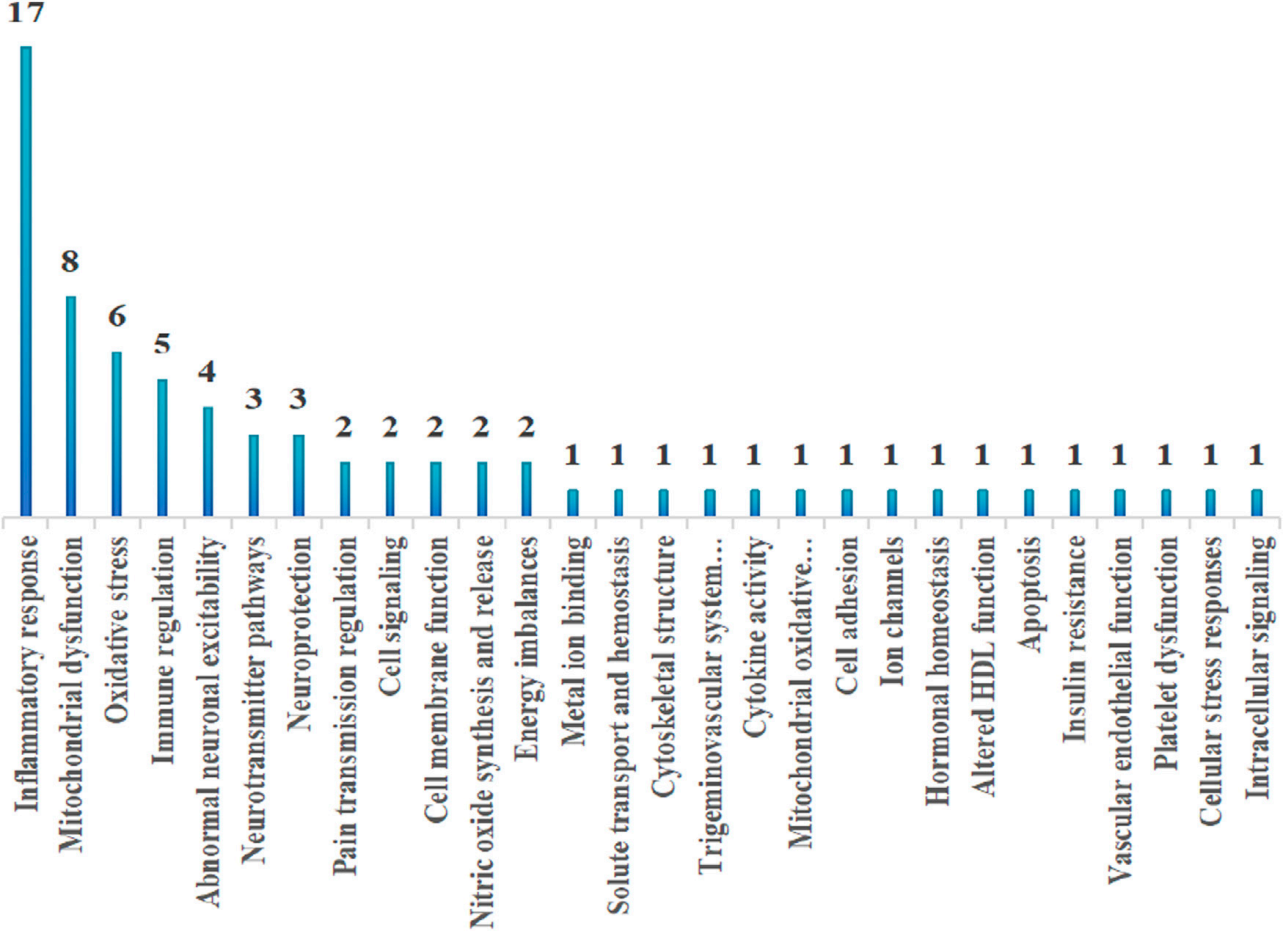
Frequency distribution of mechanisms.

By summarizing the multi-omics characteristics of different migraine subtypes and analyzing the frequency distribution of these mechanisms, we found that inflammatory responses, mitochondrial dysfunction, and oxidative stress are the most common shared mechanisms across all migraine subtypes. The frequency distribution of these mechanisms is illustrated in Figure 2 and Table 5.

**TABLE 5:** Frequency distribution of mechanisms across different omics types.

Mechanism	Genomics	Transcriptomics	Proteomics	Metabolomics	Total
Inflammatory response	5	4	4	4	17
Mitochondrial dysfunction	4	0	0	4	8
Oxidative stress	0	1	2	3	6
Immune regulation	1	1	3	0	5
Abnormal neuronal excitability	1	0	0	3	4
Neurotransmitter pathways	2	1	0	0	3
Neuroprotection	0	0	2	1	3
Pain transmission regulation	1	0	0	1	2
Cell signaling	1	1	0	0	2
Cell membrane function	0	0	1	1	2
Nitric oxide synthesis and release	0	0	0	2	2
Energy imbalances	0	0	0	2	2
Metal ion binding	1	0	0	0	1
Solute transport and hemostasis	1	0	0	0	1
Cytoskeletal structure	1	0	0	0	1
Trigeminovascular system function	1	0	0	0	1
Cytokine activity	1	0	0	0	1
Mitochondrial oxidative phosphorylation	1	0	0	0	1
Cell adhesion	0	1	0	0	1
Ion channels	0	1	0	0	1
Hormonal homeostasis	0	1	0	0	1
Altered HDL function	0	0	0	1	1
Apoptosis	0	0	0	1	1
Insulin resistance	0	0	0	1	1
Vascular endothelial function	1	0	0	0	1
Platelet dysfunction	1	0	0	0	1
Cellular stress responses	1	0	0	0	1
Intracellular signaling	1	0	0	0	1

## 4 Discussion

This systematic review summarizes the application of multi-omics approaches in elucidating the pathology of migraine. Omics data have facilitated the understanding of the complex mechanisms of migraine. Current findings indicate significant differences in genes, lncRNAs, miRNAs, proteins, and metabolites between migraine patients and healthy controls. These differences are closely related to inflammation, mitochondrial dysfunction, oxidative stress, immune regulation, increased neuronal excitability, neurotransmitter pathway abnormalities, and pain transmission regulation. Notably, frequency analysis of multi-omics mechanisms reveals that inflammation, mitochondrial dysfunction, and oxidative stress are the primary mechanisms of migraine pathogenesis. Research using various detection techniques supports these findings. For instance, Arão Belitardo Oliveira (Oliveira et al., 2017) used ELISA to detect significantly elevated levels of TNF-αand IL-12p70 in the plasma of migraine patients, indicating inflammation. Togha, Mansoureh (Togha et al., 2019), using colorimetric and photometric methods, found significantly increased levels of malondialdehyde and nitric oxide and significantly decreased activities of catalase and superoxide dismutase in the plasma of migraine patients, indicating increased oxidative stress. Sangiorgi et al. (1994), using spectrophotometry and fluorometry, found significantly reduced mitochondrial enzyme activity in the platelets of migraine patients, suggesting a close relationship between migraine occurrence and mitochondrial dysfunction.

Additionally, large-sample meta-analyses support the findings of this study. For instance, (Gormley et al., 2016). Conducted a genetic meta-analysis involving 375,000 individuals and identified 38 genetic loci associated with migraine. Among these, REST, GJA1, YAP1, PRDM16, LRP1, and MRVI1 were closely linked to oxidative stress, thus providing evidence for the oxidative stress mechanisms explored in this multi-omics analysis. Furthermore, the subtype analysis in this meta-analysis concluded that migraine without aura (MO) was significantly associated with seven genetic loci: near TSPAN2, TRPM8, PHACTR1, FHL5, ASTN2, FGF6, and LRP1. In contrast, no significant genetic loci were identified for migraine with aura (MA). The current study’s results also suggest that susceptibility genes for MO include TRPM8 and LRP1; however, the limited identification of other loci may be due to insufficient sample size.In a separate meta-analysis, Chaofan Geng (Geng et al., 2022). included 10 studies with a total of 1842 participants, revealing significant elevations in serum CRP, IL-1β, IL-6, and TNF-ɑ levels among migraine patients, thus supporting the inflammation-related mechanisms proposed in this study. While inflammation, oxidative stress, and mitochondrial dysfunction are recognized mechanisms in migraine pathophysiology, other studies have proposed various alternative mechanisms. Nonetheless, this study primarily aims to explore the main migraine mechanisms through a multi-omics approach.

Notably, in 2022, Hautakangas (Hautakangas et al., 2022) conducted a genome-wide association study (GWAS) involving 102,084 migraine patients, identifying 123 risk loci associated with migraine. This study represents one of the most comprehensive and in-depth investigations of migraine risk genes to date, providing a robust complement to our systematic review’s genomic exploration of migraine-specific loci. Our review particularly emphasizes the critical roles of mitochondrial dysfunction, inflammatory response, and oxidative stress in migraine, which may be regulated at the genetic level by the risk loci identified by Hautakangas. Consistent with their findings, our review also highlights the role of genes such as TNF-α, COX1, COX2, and COX3 in the regulation of inflammatory responses. Additionally, the Hautakangas study identified risk loci associated with mitochondrial function and oxidative stress, such as MRPS21, which encodes a component involved in mitochondrial protein synthesis and regulates mitochondrial function (Greber and Ban, 2016), and SELENBP1, which modulates oxidative stress responses (Jia et al., 2024). Although our review did not specifically identify MRPS21 and SELENBP1, functional clustering analysis in our study revealed a close association between migraine and mitochondrial dysfunction and oxidative stress, with corresponding support from transcriptomic, proteomic, and metabolomic data.

Furthermore, in 2024, the Hautakangas (Hautakangas et al., 2024) team conducted a meta-analysis of three GWAS studies on migraine, confirming multiple risk loci, including TNF-α, MRPS21, and SELENBP1, which are closely linked to inflammatory responses, mitochondrial function, and oxidative stress. Through fine-mapping of the meta-analysis results, 181 candidate causal variants were identified, including variants associated with mitochondrial function and inflammation, such as rs10218452 in PRDM16, which promotes mitochondrial biogenesis and maintenance (Harms et al., 2014), and rs9349379 in PHACTR1, which regulates inflammation and participates in oxidative stress responses (Zhang et al., 2018). This study significantly enhances our understanding of the pathogenic roles of specific genetic variants in migraine, further supporting the critical roles of mitochondrial dysfunction, inflammation, and oxidative stress in migraine, as highlighted in our review, while also supplementing our findings with detailed information on specific variant loci.

Although inflammation plays a crucial role in the pathogenesis of migraine, we cannot assess the correlation between peripheral and central inflammation levels. However, related studies (Burstein et al., 1998) indicate that peripheral inflammatory signals are received by the trigeminal ganglion, which transmits these signals to higher-order neurons in the trigeminal nucleus caudalis for head pain perception. Oxidative stress can damage vascular endothelial cells, leading to endothelial dysfunction and impaired vasomotor regulation, increasing vascular permeability, and inducing migraine (Tietjen, 2009; Sies, 2015). Mitochondrial energy metabolism disorders result in insufficient energy supply, making nerve cells more prone to fatigue and damage, thereby lowering the pain threshold and triggering migraine (Sparaco et al., 2016; Wang et al., 2023).

There are existing treatments targeting inflammation, mitochondrial dysfunction, and oxidative stress mechanisms in migraine. Non-steroidal anti-inflammatory drugs are currently recommended as first-line treatments for migraine attacks (Marchi et al., 2023), inhibiting inflammatory responses but causing gastrointestinal reactions and bleeding (Suthisisang et al., 2010; Rabbie et al., 2013; Saguil and Herness, 2014). Antioxidants such as feverfew extract (MIG-99) (Sprenger et al., 2018) and coenzyme Q10 (Colombo et al., 2014), which regulate mitochondrial energy metabolism, have shown some preventive effects for migraine, but clinical studies are limited, and there is a lack of high-quality evidence supporting their inclusion in migraine prevention guidelines. Future research should further explore the specific pathways of inflammation, oxidative stress, and mitochondrial dysfunction in migraine pathogenesis to guide clinical drug development and expand the range of pharmacological treatments for migraine.

Among the 38 studies included in this review, one genomic and two metabolomic studies reported negative results, indicating no significant differences in genes and metabolites between migraine patients and healthy controls. This contradicts other research findings. Possible reasons include a small sample size leading to insufficient statistical power to detect subtle differences in gene expression or metabolite levels. Additionally, stringent multiple testing corrections may filter out some potentially significant genes, and the two metabolomic studies used targeted approaches that only analyzed a subset of known metabolites, possibly missing important differences. Finally, variability in biological sample handling could also contribute to these negative results.

Although we categorized migraine subtypes and attempted to summarize subtype-specific mechanisms using multi-omics high-throughput technologies, no specific mechanisms were identified among the subtypes. Despite having distinct clinical features, all subtypes meet the diagnostic criteria for migraine, indicating a high degree of commonality in their underlying pathophysiological mechanisms. These commonalities are pervasive across subtypes. The databases used for biochemical analysis might only contain partial expression data and may not cover all potentially relevant biomarkers, limiting the detection and analysis of subtype-specific mechanisms.

## 5 Strengths and limitations

This study has both strengths and limitations. Our strengths lie in the comprehensive summary of findings from various omics approaches, including genomics, transcriptomics, proteomics, and metabolomics. This multi-faceted analysis enhances the breadth of understanding of the complex pathological processes underlying migraine. Additionally, we have elucidated the significant associations between inflammation, mitochondrial dysfunction, and oxidative stress in the occurrence of migraine. Lastly, the inclusion of primarily clinical observational studies, as opposed to those relying solely on animal models or primary cell cultures, ensures that our findings are more reflective of real clinical scenarios, thereby increasing their clinical relevance and practicality.

We incorporated multi-omics studies on migraine to explore its complex pathological basis. Despite the substantial and multidimensional pathology information provided by this approach, the study has notable limitations. Firstly, there is significant heterogeneity among the included studies, including differences in population characteristics and the omics methodologies used. This substantial heterogeneity prevents us from conducting a meta-analysis. Secondly, multi-omics data is insufficient in elucidating the specific mechanisms of inflammation, mitochondrial dysfunction, and oxidative stress in migraine. Lastly, the inherent drawbacks of multi-omics approaches cannot be ignored. Data standardization varies across laboratories, complicating integration and comparison. Different omics platforms may have varying sensitivity and specificity, leading to platform biases. Multi-omics research requires interdisciplinary collaboration among biologists, statisticians, and computational scientists, posing coordination challenges. Additionally, the large and complex nature of multi-omics data makes storage and sharing difficult.

## 6 Future directions

Future research should focus on several key areas. Firstly, it is essential to increase the diversity and representativeness of samples, including a broader population and clearly defined migraine subtypes, to deeply explore the specific pathological mechanisms of each subtype. Additionally, further experimental validation and larger-scale studies are needed to improve the reliability and generalizability of omics research results. Finally, establishing standardized processes for data generation and analysis will facilitate data integration and comparison. Implementing calibration techniques to align the sensitivity and specificity of different omics platforms will reduce platform biases. Enhancing interdisciplinary collaboration and developing advanced data management systems are crucial to address the challenges in the implementation of multi-omics approaches.

## 7 Conclusion

In summary, this systematic review comprehensively integrates and analyzes clinical multi-omics research findings on migraine, demonstrating that the pathogenesis of migraine involves complex Pathological processes, including genetic variations, transcriptional abnormalities, protein function alterations, and metabolic imbalances. We particularly emphasize the central roles of mitochondrial dysfunction, inflammatory responses, and oxidative stress in disease progression. The integration of multi-omics studies enhances the understanding of the Pathological basis of migraine and provides potential avenues for developing new diagnostic criteria and therapeutic targets.

## Data Availability

The original contributions presented in the study are included in the article/Supplementary Material, further inquiries can be directed to the corresponding authors.

## Abbreviations

H, Healthy Control Group; MICP, Migraine Ictal Phase; MINP, Migraine Interictal Phase; MA,migraine with aura; MO, Migraine without aura; CM, chronic migraine; MM, menstrual-related migraine; CSF, Cerebral spinal fluid; 1H-NMR, Nuclear Magnetic Resonance; UPLC-MS, ultra-performance liquid chromatography-mass spectrometry; HPLC-ESI-MS, High Performance Liquid Chromatography - Electrospray Ionization - Mass Spectrometry; GC, Gas Chromatography; 2DE, two-dimensional gel electrophoresis; LC-MS, Liquid Chromatograph Mass Spectrometer; MS, mass spectrometry; LC-ESI-QToF-MS, Liquid Chromatography - Electrospray Ionization - Quadrupole Time of Flight - Mass Spectrometry; MALDI-TOF-MS, matrix-assisted laser desorption/ionization time-of-flight mass spectrometry; PBL, peripheral blood lymphocytes; WB, Whole blood; NSM, non-subtyped migraine; ROS, reactive oxygen species; 31 NMR, 31 nuclear magnetic resonance; PCr, phosphocreatine; Pi, phosphate.
