# Supplementary material for: Multi-omics approaches to deciphering complex pathological mechanisms of migraine: a systematic review
Source: Front Pharmacol. 2025 Jan 9;15:1452614. doi: 10.3389/fphar.2024.1452614 (PMC11754399; doi:10.3389/fphar.2024.1452614)
Supplement: Supplementary file 1 [file Table1.docx]

| **NO.** | **Search items** | **NO.** | **Search items** |
| --- | --- | --- | --- |
| 1 | Genomics | 35 | Headache, Migraine |
| 2 | Comparative Genomics | 36 | Headaches, Migraine |
| 3 | Comparative Genomic | 37 | Migraine Headaches |
| 4 | Genomic, Comparative | 38 | Status Migrainosus |
| 5 | Genomics, Comparative | 39 | Hemicrania Migraine |
| 6 | Metabolomics | 40 | Hemicrania Migraines |
| 7 | Metabolomic | 41 | Migraine, Hemicrania |
| 8 | Metabonomics | 42 | Migraines, Hemicrania |
| 9 | Metabonomic | 43 | Sick Headache |
| 10 | Epigenomics | 44 | Migraine without Aura |
| 11 | Epigenomic | 45 | Common Migraines |
| 12 | Epigenetics | 46 | Common Migraine |
| 13 | Epigenetic | 47 | Migraines, Common |
| 14 | Glycomics | 48 | Migraine, Common |
| 15 | Glycobiology | 49 | Migraine with Aura |
| 16 | Proteomics | 50 | Migraine with Auras |
| 17 | Lipidomics | 51 | Migraine with Typical Aura |
| 18 | Lipidomic | 52 | Classical Migraine |
| 19 | Lipidome | 53 | Migraine, Classical |
| 20 | Lipidomes | 54 | Migraine, Prolonged Aura |
| 21 | Transcriptomics | 55 | Prolonged Aura Migraine |
| 22 | Spatial transcriptomics | 56 | Migraine, Classic |
| 23 | Omics | 57 | Classic Migraine |
| 24 | Multi-omics | 58 | Migraine with Acute Onset Aura |
| 25 | 1 OR 2-24 | 59 | Acute Onset Aura Migraine |
| 26 | Migraine Disorders | 60 | Migraine with Auras |
| 27 | Disorders, Migraine | 61 | Migraine with Prolonged Aura |
| 28 | Migraine Disorder | 62 | Migraine Aura without Headache |
| 29 | Headache, Sick | 63 | Typical Aura without Headache |
| 30 | Headaches, Sick | 64 | Chronic Migraine |
| 31 | Sick Headaches | 65 | Episodic Migraine |
| 32 | Migraine | 66 | Menstrual Migraine |
| 33 | Migraines | 67 | 26 or 27-66 |
| 34 | Migraine Headache | 68 | 25 and 67 |

**Supplementary Table 1.** Search Strategy Example: PubMed search
