# Supplementary material for: Multi-omics approaches to deciphering complex pathological mechanisms of migraine: a systematic review
Source: Front Pharmacol. 2025 Jan 9;15:1452614. doi: 10.3389/fphar.2024.1452614 (PMC11754399; doi:10.3389/fphar.2024.1452614)
Supplement: Supplementary file 2 [file Table2.docx]

**Supplementary Table 2** Evaluation of methodological quality of included cross-sectional studies

| **Study** | **Define the source of informa-tion** | **List inclusion and exclusion criteria for exposed and unexposed subjects (cases and controls) or refer to previous publications** | **Indicate time period used for identifying patients** | **Indicate whether or not subjects were consecutive if not population-based** | **Indicate if evaluators of subjective components of study were masked to other aspects of the status of the participants** | **Describe any assessments undertaken for quality assurance purposes** | **Explain any patient exclusions from analysis** | **Describe how confounding was assessed and/or controlled** | **If applicable, explain how missing data were handled in the analysis** | **Summarize patient response rates and completeness of data collection** | **Clarify what follow-up, if any, was expected and the percentage of patients for which incomplete data or follow-up was obtained** | **Total score**  **（No or Unclear = 0, Yes = 1）** |
| --- | --- | --- | --- | --- | --- | --- | --- | --- | --- | --- | --- | --- |
| [Rustichelli et al., 2021](#_ENREF_58) | Yes | Yes | Yes | Yes | Unclear | Yes | No | Unclear | No | Yes | Unclear | 6 |
| [K M Welch, 1989](#_ENREF_34) | Yes | No | No | No | Unclear | No | No | Yes | No | Yes | Unclear | 3 |
| [Onderwater et al., 2023](#_ENREF_46) | Yes | Yes | No | Unclear | Unclear | No | Yes | Unclear | Yes | Yes | Unclear | 5 |
| Shang et al,  2012 | Yes | Yes | Ys | Yes | Unclear | No | No | Unclear | No | Yes | Unclear | 5 |
| [Rustichelli et al., 2020](#_ENREF_57) | Yes | Yes | No | Yes | Unclear | No | No | Unclear | No | Yes | Unclear | 4 |
| [Peterlinet al., 2015](#_ENREF_48) | Yes | Yes | Yes | Yes | Unclear | No | No | Unclear | No | Yes | Unclear | 5 |
| [Bellei et al., 2021](#_ENREF_8) | Yes | Yes | No | Yes | Unclear | No | No | Unclear | No | Yes | Unclear | 4 |
| [Bellei et al., 2020](#_ENREF_9) | Yes | Yes | No | Yes | Unclear | No | No | Unclear | No | Yes | Unclear | 4 |

**Table 3** Evaluation of methodological quality of included case control studies

| **Study** | **Is the case adequate definition** | **Representativeness of the cases** | | **Selection of Controls** | **Definition of Controls** | **Comparability of cases and controls on the basis of the design or analysis** | **Ascertainment of exposure** | **Same method of ascertainment for cases and controls** | **Non-Response rate** | **Total score**  **（*= 1）** |
| --- | --- | --- | --- | --- | --- | --- | --- | --- | --- | --- |
| [Ronald](https://pubmed.ncbi.nlm.nih.gov/?term=Zielman+R&cauthor_id=27734045) [et al, 2016](#_ENREF_51) | * | 0 | 0 | | * | 0 | 0 | 0 | 0 | 2 |
| [Ren et al., 2018a](#_ENREF_52) | * | * | 0 | | * | ** | 0 | * | 0 | 6 |
| [Curto et al., 2015](#_ENREF_17) | * | 0 | 0 | | * | * | 0 | 0 | 0 | 3 |
| [Harder et al., 2021](#_ENREF_29) | * | * | * | | 0 | ** | 0 | * | 0 | 6 |
| [Onderwater et al., 2019](#_ENREF_45) | * | * | * | | 0 | ** | 0 | * | 0 | 6 |
| [Aczél et al., 2021](#_ENREF_3) | * | 0 | 0 | | * | * | 0 | 0 | 0 | 3 |
| [de Tommaso et al., 2012](#_ENREF_19) | * | * | * | | 0 | * | 0 | 0 | 0 | 4 |
| [D'Andrea et al., 2022](#_ENREF_18) | * | * | 0 | | 0 | * | 0 | 0 | 0 | 3 |
| [Ren et al., 2018b](#_ENREF_53) | * | 0 | 0 | | * | * | 0 | 0 | 0 | 3 |
| [Gouveia-Figueira et al., 2017](#_ENREF_25) | * | * | 0 | | 0 | * | 0 | 0 | 0 | 3 |
| [Tuka et al., 2021](#_ENREF_65) | * | 0 | 0 | | 0 | ** | 0 | 0 | 0 | 3 |
| [Lionetto et al., 2021](#_ENREF_39) | * | 0 | 0 | | * | * | 0 | 0 | 0 | 3 |
| [Togha et al., 2022](#_ENREF_63) | * | * | * | | * | * | 0 | * | 0 | 5 |
| [Guyuron et al., 2014](#_ENREF_28) | * | * | 0 | | * | ** | * | * | 0 | 7 |
| [Xu et al., 2022](#_ENREF_70) | * | 0 | 0 | | * | ** | 0 | 0 | 0 | 4 |
| [Aczél et al., 2022](#_ENREF_2) | * | * | 0 | | * | ** | 0 | 0 | 0 | 5 |
| [Lin et al., 2020](#_ENREF_38) | * | * | 0 | | 0 | 0 | 0 | 0 | 0 | 2 |
| [Gallardo et al., 2023](#_ENREF_21) | * | * | 0 | | * | ** | 0 | 0 | 0 | 5 |
| Andersen [et al., 2016](#_ENREF_5) | * | * | 0 | | 0 | 0 | 0 | 0 | 0 | 2 |
| [Pan et al., 2019](#_ENREF_47) | * | 0 | 0 | | 0 | 0 | 0 | 0 | 0 | 1 |
| [Hershey et al., 2012](#_ENREF_31) | * | * | 0 | | * | ** | 0 | * | 0 | 6 |
| [Gerring et al., 2018](#_ENREF_22) | * | * | 0 | | 0 | * | 0 | 0 | 0 | 3 |
| [Hershey et al., 2004](#_ENREF_32) | * | * | 0 | | * | ** | 0 | 0 | 0 | 5 |
| [Al Asoom et al., 2022](#_ENREF_4) | * | * | * | | * | * | 0 | * | 0 | 6 |
| Chasman [et al., 2011](#_ENREF_13) | * | * | 0 | | * | 0 | 0 | * | 0 | 4 |
| Freilinger et al., 2012 | * | * | * | | * | 0 | 0 | * | 0 | 5 |
| [Chen et al., 2017](#_ENREF_14) | * | * | 0 | | * | * | 0 | 0 | 0 | 4 |
| Khan [et al., 2022](#_ENREF_35) | * | * | * | | * | ** | 0 | * | 0 | 7 |
| [Nagata et al., 2009](#_ENREF_43) | * | 0 | 0 | | * | * | 0 | 0 | 0 | 3 |
| [Kogelman et al., 2019](#_ENREF_36) | * | * | * | | 0 | 0 | 0 | * | 0 | 4 |
| [Carreño et al., 2011](#_ENREF_11) | * | * | * | | * | * | 0 | 0 | * | 6 |
